# Instruments to assess suicide risk: a systematic review

**DOI:** 10.1590/2237-6089-2019-0092

**Published:** 2020-10-08

**Authors:** Ezequiel T. Andreotti, Jaqueline R. Ipuchima, Silvio César Cazella, Pedro Beria, Cristiane Flôres Bortoncello, Richard Chuquel Silveira, Ygor Arzeno Ferrão

**Affiliations:** 1 Universidade Federal de Ciências da Saúde de Porto Alegre Porto AlegreRS Brazil Universidade Federal de Ciências da Saúde de Porto Alegre (UFCSPA), Porto Alegre , RS , Brazil .

**Keywords:** Scale, suicide, assessment, questionnaire

## Abstract

**Introduction:**

Suicide is an issue of great severity in public health worldwide. This study aimed to investigate which instruments are most frequently used by healthcare professionals to assess suicide risk and how accessible such instruments are, as well as to determine the scope of suicide phenomena.

**Method:**

A systematic review was performed using the following Boolean searches: “scale AND suicide,” “evaluation AND suicide,” “questionnaire AND suicide.” The articles retrieved were read and selected by two independent researchers – any discrepancies were addressed by a third researcher.

**Results:**

From a total number of 206 articles, 20 instruments were identified as being currently used to assess suicide risk. The two most common were the Beck Scale for Suicide Ideation (BSI) and The Columbia – Suicide Severity Rating Scale (C-SSRS).

**Conclusion:**

Even though the two scales (BSI and C-SSRS) are the most frequently mentioned and used by healthcare professionals to assess suicide risk, both instruments present breaches in their structure and there is not yet a single instrument considered to be the gold standard. As a future perspective, there is the urgency of developing a new tool that can widely and completely assess all psychopathological aspects of suicidality.

## Introduction

Suicide is an issue of great severity in public health all over the globe. It can be understood as self-inflicted violence in which the individual tries to end their own life due to a series of factors related to biological, psychological and environmental issues. ^1^ Suicidality, in turn, is assumed to be the group of suicidal thoughts, but it also encompasses the planning and the suicide attempt among other related aspects leading to the suicide act. ^2^ The World Health Organization (WHO) reported an increase of 60% in suicide episodes in the last 45 years, ^3^ with about 800 thousand deaths resulting from it. It was also estimated that by 2020 this number would reach 1.5 million people, which means there will be a death every 40 seconds as a result of suicide attempt. ^4^

Due to the severity of this topic, the WHO has been publishing guide books for healthcare professionals in order to allow them to create strategies to help prevent suicide. These materials have been addressed to medical practitioners, media professionals, as well as teachers and other educators. ^5^ In 2006, the Brazilian Ministry of Health developed a suicide preventive guide book for healthcare professionals in which information about suicide was made available, as well as instructions to help these professionals identify and deal with individuals at imminent risk of suicide. ^6^ In 2014, the Brazilian Psychiatric Association and the Federal Council of Medicine worked together to develop and publish the guide book titled *Suicídio: informando para prevenir* (in English, *Suicide: informing to prevent* ). ^7^

Suicide is considered a complex subject and a multifactorial phenomenon that can be attempted by an individual both in purposely planned environments and in supposedly protected places (such as hospital settings and health clinics). However, when a suicide episode occurs within the hospital environment, its consequences reach beyond the victim and their family, also affecting the healthcare professionals in that setting. Due to their work routines, these professionals are prone to face suicidality, ^8^ and despite the prevalence of the phenomenon in this field, some professionals might not feel confident or capable to detect, prevent and manage patients. Therefore, the identification and dimensioning of suicide and suicidality is considered to be extremely relevant, especially with the use of instruments capable to make the assessment of the suicidal individual by the healthcare professionals more objective and accurate.

Some score scales tend to be widely used by mental health professionals, especially psychiatrists and psychologists, to evaluate the aspects related to suicide – such as impetus, planning, intention, previous attempts, and others. Two of these instruments are the Beck Scale for Suicide Ideation (BSI) ^9^ and The Columbia – Suicide Severity Rating Scale (C-SSRS). ^10^ However, as previously stated in a systematic review about suicide risk, there is a variety of at least 19 instruments for assessing suicide risk. Many of these instruments are known to have accessibility problems (e.g., high financial costs, long and hard to administer, lack of psychometric evidence). ^11^

Because we consider this to be a topic of great matter, this systematic review aimed to investigate which instruments are most frequently used by healthcare professionals to assess suicide risk and also how accessible these instruments are.

## Method

This systematic review followed the Preferred Reporting Items for Systematic Reviews and Meta-Analyses (PRISMA) ^12^ recommendations and was based on the following Boolean searches: “scale AND suicide,” “evaluation AND suicide,” “questionnaire AND suicide.” All articles inserted in the database were arranged by one of this study’s researchers. Two independent researchers conducted the searches and applied inclusion/exclusion criteria in February 2018.

The inclusion criteria were: articles published in the last 10 years (2008-2018) in the PubMed database; and articles published in Portuguese, English or Spanish involving humans. Articles that did not use instruments to assess suicide risk as a central subject were excluded (this was the only exclusion criterion). Because the aim was to identify the instruments most frequently used, a box plot graph was designed to enable easy visualization of the results found. A detailed analysis (construct validity, reliability and applicability) was performed in those instruments considered to be the median outliers.

Literature search and selection was conducted by three researchers; two of them acted as reviewers in an independent and blinded way (ETA and JRI). First, a search was conducted in the PubMed MEDLINE database. All articles and abstracts initially retrieved were analyzed and selected whenever appropriate. Both reviewers used the same inclusion and exclusion criteria mentioned above and compared the results with one another. Any discrepancies originated during the process of analysis and selection were resolved by a third researcher (PB).

## Results

**Figure 1:**
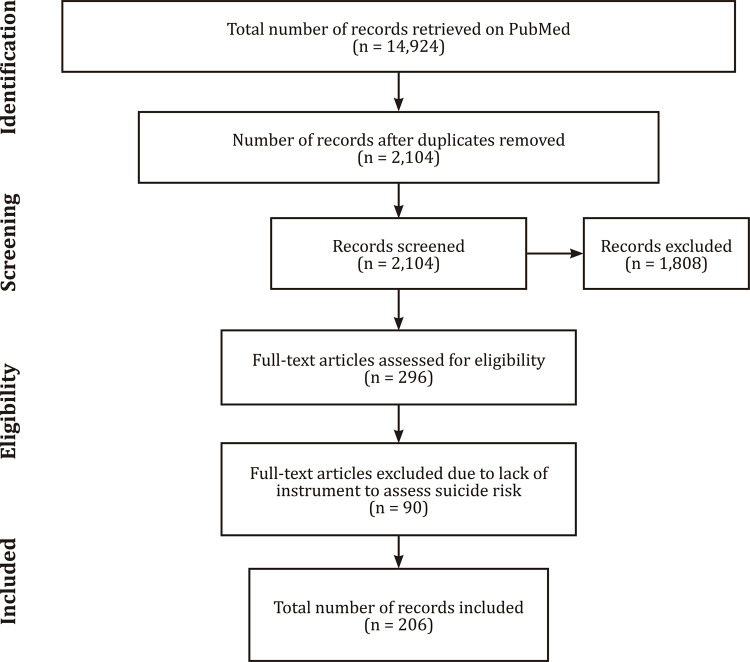
Flowchart illustrating study selection according to the Preferred Reporting Items for Systematic Reviews and Meta-Analyses (PRISMA).

We first retrieved 14,924 papers. After applying the inclusion and exclusion criteria, this number reduced to 206. Figure 1 and Table 1 show these results.

**Table 1:** Articles resulting from the Boolean searches, inclusion and exclusion criteria applied (phase 1), and discrepancies resolved by a third researcher (phase 2)

Phase 1: Performed by R1 and R2								Phase 2: Discrepancies resolved by R3					

			Articles after exclusion criteria (n = 296)			Discrepancies in selection R1 × R2		Discrepancy solution by R3 (n = 214)		Total articles after R3 intervention			

Boolean search	Articles retrieved (n)	Articles after inclusion criteria	R1 (n = 180; 60.8%)	R2 (n = 198; 66.9%)	Articles in common R1/R2	R1	R2	R1	R2	Common	R1	R2	Total
“scale AND suicide”	3,579	481 (13.4)	44/481 (9.1)	52/481 (10.8)	22/52 (42.3)	22/52 (42.3)	30/52 (57.7)	15/22 (68.2)	16/30 (53.3)	22	15	16	53
“evaluation AND suicide”	4,232	525 (12.4)	44/525 (8.4)	47/525 (8.5)	15/61 (24.6)	29/47 (61.7)	32/47 (68.1)	27/29 (93.1)	28/32 (67.5)	15	27	28	70
“questionnaire AND suicide”	7,113	1,098 (15.4)	92/1,098 (8.4)	99/1,098 (9.0)	45/101 (44.6)	47/99 (47.5)	54/99 (54.5)	19/37 (51.4)	19/42 (45.2)	45	19	19	83
Total					82/296 (27.7)	98/296 (33.1)	116/296 (39.2)	61/86 (70.9)	63/104 (60.6)	82	61	63	**206**
						Total discrepancies: 214 (72.3)		Total: 124/214 (57.9%)					

Results presented as n (%), unless otherwise specified.

R1, R2, R3 = Researcher 1, 2, 3.

**Figure 2:**
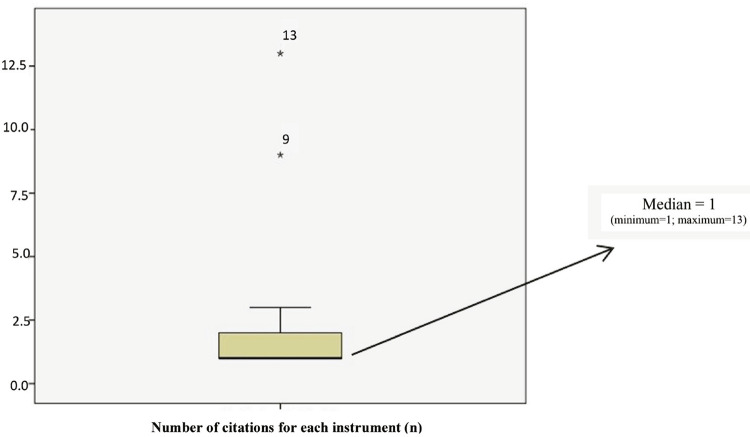
Box plot graph showing frequency of citation of the suicide assessment instruments. *9 = The Columbia – Suicide Severity Rating Scale. *13 = Beck Scale for Suicide Ideation.

Among the 206 articles included in the study, we identified 20 instruments used by healthcare professionals to assess and prevent suicide risk. These included: the BSI, mentioned 13/206 (6.31%) times; the C-SSRS, mentioned 9/206 (4.36%) times; the Beck Suicidal Intent Scale (SIS), mentioned 3/206 (1.5%) times; the Paykel Suicide Scale, mentioned 2/206 (0.97%) times; the Suicidal Ideation Questionnaire-Junior (SIQ-JR), mentioned 2/206 (0.97%) times; the Beck Suicide Scale – worst ever version (BSSw), mentioned 2/206 (0.97%) times; the Suicidal Ideation Questionnaire (SIQ), mentioned 2/206 (0.97%) times; the Mini-International Neuropsychiatric Interview (MINI), mentioned 2/206 (0.97%) times; The asQ’em Screening Instrument, mentioned 1/206 (0.49%) times; the Scale of Public Attitudes about Suicide (SPAS), mentioned 1/206 (0.49%) times; the Risk of Suicide Questionnaire – Revised (RSQ-R), mentioned 1/206 (0.49%) times; The Risk of Suicide Questionnaire (RSQ), mentioned 1/206 (0.49%) times; the Suicide Score Scale (SSS), mentioned 1/206 (0.49%) times; the Suicide Opinion Questionnaire (SOQ), mentioned 1/206 (0.49%) times; the WMH Composite International Diagnostic Interview (WMH-CIDI), mentioned 1/206 (0.49%) times; the Intersept Suicide Scale (ISST), mentioned 1/206 (0.49%) times; the Plutchik Suicide Risk Scale mentioned 1/206 (0.49%) times; the Chinese shortened version of the SIS (C-SIS), mentioned 1/206 (0.49%) times; the Harkavy-Asnis Suicide Scale (HASS), mentioned 1/206 (0.49%) times; and the Suicide Probability Scale (SPS), mentioned 1/206 (0.49%) times. Figure 2 shows two instruments as median outliers, namely, BSI and C-SSRS.

Summarized data related to the two most frequently cited scales are shown in Table 2 .

**Table 2:** Assessment and prevention instruments most frequently cited in the literature used to identify and measure suicidality and suicide risk

Instrument	Characteristics	Reference	No. papers
Beck Scale for Suicide Ideation (BSI)	The original BSI was developed in 1988, modeled after a successful interviewer-rated version, the Scale for Suicide Ideation. ^9^ The BSS contains 19 items that measure the severity of actual suicidal wishes and plans. Scores may range from 0 to 38, with a higher score indicating a higher level of suicide ideation.	Beck et al. ^9^	13/206 (6.31%)
The Columbia – Suicide Severity Rating Scale (C-SSRS)	The C-SSRS consists of 18 items and has been shown to predict suicide attempts in both suicidal and non-suicidal individuals. It was developed as a brief 4-item measure to assess potential suicide risk, in consideration of competing demands in medical practice that may hinder the use of longer scales. The C-SSRS, however, was designed to distinguish between the domains of suicidal ideation and suicidal behavior. Four constructs are measured. The first is the severity of ideation (hereafter referred to as the severity subscale), which is rated on a 5-point ordinal scale where 1=wish to be dead, 2=nonspecific active suicidal thoughts, 3=suicidal thoughts with methods, 4=suicidal intent, and 5=suicidal intent with plan. The second subscale assesses the intensity of ideation (hereafter referred to as the intensity subscale), which comprises 5 items, each rated on a 5-point ordinal scale, covering frequency, duration, controllability, deterrents, and reason for ideation. The third is the behavior subscale, rated on a nominal scale that includes actual, aborted, and interrupted attempts, preparatory behavior, and non-suicidal self-injurious behavior. And the fourth is the lethality subscale, which assesses actual attempts; actual lethality is rated on a 6-point ordinal scale: if actual lethality is zero, potential lethality of attempts is rated on a 3-point ordinal scale.	Posner et al. ^10^	9/206 (4.36%)

## Discussion

Despite the small number of articles included in the analysis (a limitation of this study), a high number of instruments (20) were found to be used to detect and assess suicide risk. However, two scales showed a substantially higher number of citations (found in over 4% of the reviewed articles), namely, the BSI and the C-SSRS. Therefore, these two scales are described in more detail below.

### The Beck Scale for Suicide Ideation (BSI)

The BSI comprises three sections that aim to assess severity of suicide ideation. The scale was created in the United States and adapted to the Mexican ^13^ and Brazilian ^14^ populations. The Brazilian Portuguese version comprises 21 items, each presenting three options that are graded from 0 to 2 in intensity; the total score may range from 0 to 38, where a higher score indicates higher suicide ideation. ^9^

The first section of the BSI presents five questions about the wish to die: 1) wish to live, 2) wish to die, 3) reasons to live, 4) wish to commit suicide, and 5) self-protection in case of a life threatening event. In this first part, when questions 4 and 5 score zero, the interviewer should skip section 2 and proceed to section 3.

Section 2, with questions from 6 to 19, focuses on suicide ideation: 6) periods of suicide thoughts, 7) suicide thoughts, 8) acceptance of the suicide ideation, 9) control over committing suicide, 10) deterrents for suicide (such as family, friends), 11) reasons to commit suicide, 12) a specific plan of how to commit suicide, 13) accessibility to a method or specific opportunity to commit suicide, 14) courage or capability to commit suicide, 15) the wait to attempt suicide, 16) preparations to commit suicide, 17) a suicide note, 18) thoughts of what should be done after suicide, and 19) hiding the wish to commit suicide from people. When section 2 questions are covered, the interviewer is directed to the next section.

The third and final section presents only two questions, related to the suicide attempt (questions 20 and 21): 20) suicide attempt, 21) intensity of the wish to die related to the suicide attempt.

Despite being widely used and considered a good reference to assess patients with suicide risk based on their wish to die, suicide ideation and suicide attempts, a breach that has been considered is the fact that the BSI is commonly applied to patients who already are at risk of suicide. It is important to mention that the BSI is widely used by healthcare professionals as a supportive tool for clinical assessment, as it comprises aspects that effectively evaluate the suicide context. It also allows doctors to choose, from a range of investigation paths, the one that will suit the assessed individual in order to deliver the best care and treatment.

### The Columbia – Suicide Severity Rating Scale (C-SSRS)

The C-SSRS is comprised of four sections presenting 18 items that aim to predict potential suicide risk in both suicidal and non-suicidal individuals. This scale was created in the United States and adapted to over 100 country-specific languages. ^10^ Many of these translations have been linguistically validated. It is worth saying that the C-SSRS was designed to differentiate suicide ideation from suicide behavior ^10^ . Its sections are described below.

Section 1 deals with the severity of ideation (severity subscale), rated from 1 to 5, where 1 = wish to be dead, 2 = non-specific active suicidal thoughts, 3 = suicidal thoughts with methods, 4 = suicidal intent, and 5 = suicidal intent with plan.

Section 2 comprises the intensity subscale, including 5 items, each of them rated on a 5-point ordinal scale: frequency, duration, controllability, deterrents, and reason of suicide ideation.

The following section consists of the behavior subscale, which is rated on a nominal scale that includes actual, aborted and interrupted suicide attempts, preparatory behavior, and non-suicidal self-injurious behavior.

The fourth and last section is considered to be the lethality subscale. This section assesses the actual lethality of suicide attempts, which is rated on a 6-point ordinal scale. When this subscale scores zero, then potential lethality is rated on a 3-point ordinal scale.

The C-SSRS scale is commonly used as a tool to anticipate suicide risk in both suicidal and non-suicidal individuals. Importantly, this scale presents well thought aspects to provide a good and accurate assessment of individuals that are or are not at risk of suicide, which makes it a good instrument for clinical assessment and enables the choice and use of effective strategies in the health field.

### Comparing the accessibility of BSI and C-SSRS

One of the differences between BSI and C-SSRS is that BSI is commonly used only in patients who are already at risk of suicide. In this sense, the C-SSRS seems to be more accessible, as it can be used in individuals considered or not to be at suicide risk, in order to assess their potential risk for suicide.

Conversely, besides being divided into four sections, the questions in the C-SSRS are longer than the ones in the BSI. The latter, in turn, is divided into three sections with more objective questions, which simplifies its use by healthcare professionals. Another important point is that the BSI can be self-administered, whereas the C-SSRS cannot.

Another highlight is the fact that the C-SSRS has been adapted to over 100 countries, and this information can be easily consulted on line, e.g., in a website. Even though the BSI is also known to have been adapted to many languages, there is no easy-to-access website available listing the countries/languages in which the BSI has been validated. This makes BSI less accessible, as it will be more difficult for healthcare professionals to find out whether the instrument has been validated in their country.

Therefore, although both scales present good psychometric evidence in their original versions and cross-cultural adaptations, the BSI appears to be easier to apply, whereas the C-SSRS is more accessible.

About the other instruments identified, one of the reasons for their low use/citation may be related to the high costs involved, especially when applied to large samples. Other main reasons could include long application times and lack or low quality of psychometric evidence. These findings are in agreement with a previous systematic review with meta-analysis. ^11^

## Conclusion

From the 20 instruments found to be used by healthcare professionals in the detection and assessment of suicide risk in the articles resulting from our PubMed database search, the BSI and the C-SSRS were the two most frequently mentioned as a standard tool for the clinical evaluation of individuals that are or are not at risk of suicide. Even though both were frequently used, the BSI was found to be easier to apply, and the C-SSRS more accessible across countries. Also, both the BSI and the C-SSRS present limitations related to the individual to be assessed, and it was not yet possible to identify a gold standard.

Through the analysis of the components of each scale, it was possible to observe that some aspects are contemplated by one or the other instrument, but are not present in both. Therefore, the development of a new tool to assess suicide risk and capable of solving the breaches identified in these scales is suggested.

As a future perspective, it is urgent to develop a new tool capable to widely and completely evaluating all psychopathological aspects of suicidality, including the wish to die, suicide ideation, suicide attempt, severity and intensity of ideation and suicidal behavior. Such instrument should encompass few sections with more specific and less extensive questions, so as to enable a better understanding for both interviewee and interviewer.
